# Morphological awareness and reading skill for deaf and hearing adults

**DOI:** 10.1093/jdsade/enaf042

**Published:** 2025-06-21

**Authors:** Emily Saunders, Haley Helms, Karen Emmorey

**Affiliations:** Joint Doctoral Program in Language and Communicative Disorders, San Diego State University/ University of California, San Diego, 5500 Campanile Dr, San Diego CA 92182, United States; School of Speech, Language, and Hearing Sciences, San Diego State University, 5500 Campanile Dr, San Diego, CA 92182, United States; School of Speech, Language, and Hearing Sciences, San Diego State University, 5500 Campanile Dr, San Diego, CA 92182, United States

## Abstract

Both deaf and hearing readers use morphological awareness skills to decode and comprehend printed English. Deaf readers, for whom phonological awareness is a relative weakness while orthographic sensitivity is a strength, may have a different relationship with morphology than similarly skilled hearing readers. This study investigated the impact of various reading sub-skills—spelling, vocabulary size, morphological awareness, and phonological awareness—on reading comprehension for deaf and hearing adult readers. Morphological awareness had a stronger relationship with reading comprehension for deaf than hearing readers, particularly for deaf readers with advanced morphological skills. Morphology and vocabulary were also more strongly related for the deaf group, indicating that deaf readers leverage morphology to expand their word knowledge. Overall, the findings highlight the unique and significant role of morphological awareness in the skilled deaf reader’s “toolbox” and underscore the importance of morphological instruction in supporting the reading development of deaf individuals.

## Introduction

Reading is a complex task that requires the coordination of multiple linguistic and cognitive skills working in concert. Typical hearing readers must recognize arbitrary symbols, connect them to the sounds of the corresponding spoken language, identify the meaning of individual words, and finally integrate them into a larger context. There are a number of sub-skills that make up the “toolbox” with which readers approach this complex linguistic task, including spelling ability, vocabulary size, and phonological awareness. Phonological awareness in particular has long been considered a vital component of successful reading for all readers. However, there exists a sizable population of readers who manage the complex linguistic task of reading without complete access to phonological information: those who were either born deaf or became deaf before acquiring spoken language. Some deaf individuals become very successful readers and read at levels equal to or surpassing their hearing peers, despite their relative weakness in phonological representations (see Emmorey & Lee, 2021, for review).

Reduced access to phonological information of the corresponding spoken language is therefore not an insurmountable obstacle to reading success. Research with adult deaf readers has aimed to characterize the unique processes by which they achieve reading proficiency, and particularly which skills are most important to those processes. Sehyr & Emmorey (2022) examined the relative contribution of various lexical quality variables for skilled adult readers, finding that while phonological awareness was a strong predictor for hearing readers, it was not a significant contributor to reading comprehension for equivalently skilled deaf readers (see also Mayberry et al., 2011). Furthermore, spelling skill was only a significant predictor for deaf readers, suggesting that orthographic representations play a stronger role in the reading process for deaf than hearing readers. The authors interpreted these results as indicating that skilled deaf readers develop precise orthographic representations of lexical items and rely on the mapping between orthography and semantics to access word meaning when reading. This interpretation implies a unique division of labor that prioritizes the orthographic and semantic components of the Seidenberg & McClelland (1989) triangle model of reading, compensating for poorer phonological representations. Other studies examining deaf adults’ reading comprehension have also indicated that phonological awareness does not significantly predict reading success, while spelling and vocabulary-related skills do (Cates et al., 2022; Clark et al., 2011), further supporting the hypothesis that the quality of orthographic and semantic representations is particularly important to reading success for deaf readers.

One sub-skill of reading that is highly related to orthographic-to-semantic mapping is morphological awareness, or the ability to identify and manipulate meaningful parts of complex words (Levesque et al., 2017). In order to effectively process multimorphemic words, skilled readers must be able to correctly decompose roots, stems, and affixes to identify the meaning of the whole word. Furthermore, readers must understand how derivational morphology changes the syntactic class of words (i.e., adding a noun suffix to an adjective or verb, resulting in a noun; *operate → operation*) and interpret clauses and phrase structure accordingly (Wilson-Fowler & Apel, 2015). As such, morphological processing recruits from multiple levels of linguistic understanding to effectively meet processing demands (Carlisle, 2003). Understanding morphological structure and the processes by which morphemes are combined in words (i.e, morphological awareness) likely plays a large role in the success of developing readers (Foorman et al., 2012; Kirby et al., 2012). Particularly for a language with an orthography as phonetically opaque as English, familiarity with morpheme boundaries and frequent affixes can aid readers in resolving ambiguities and determining the correct pronunciation of new words (Rastle, 2019). Indeed, the spelling-to-meaning mapping of morphemes is often more consistent than the spelling-to-sound mapping (Berg & Aronoff, 2017).

Furthermore, morphological awareness is also known to be a compensatory factor for hearing readers with phonological deficits such as dyslexia (Farris et al., 2021; Haft et al., 2016), possibly due to the fact that the English morphological system is orthographically regular and less dependent on phonology. Particularly once readers pass a middle school reading level, where the majority of words encountered are morphologically complex (Nagy & Anderson, 1984), morphological awareness may become more important to word reading and overall comprehension as readers use known morphemes as a guide to infer meanings of new words (Levesque et al., 2017). While most studies use morphological awareness measures to predict reading comprehension, Kotzer et al. (2021) investigated the reading sub-skills that contribute to morphological awareness. For college students (all hearing skilled readers) vocabulary and spelling skills were both found to significantly predict morphological awareness. This result emphasizes the way in which morphology recruits both orthographic and semantic representations, and skilled reading involves the successful integration of both. Kotzer et al. (2021) also found that phonological decoding skill (identifying pseudohomophones) was a significant but weaker predictor of morphological awareness.

Both deaf and hearing readers take advantage of morphological awareness to access the meaning of complex words, with several studies finding links between students’ awareness of morphological structure and their performance on word decomposition and decoding tasks (Carlisle, 2000; Deacon et al., 2014; Trussell & Easterbrooks, 2017; Wilson-Fowler & Apel, 2015; Kotzer et al., 2021). Overall, however, reading outcomes remain highly variable for deaf students. Deaf students often test at reading levels below their hearing peers (Kelly & Barac-Cikoja, 2007; Traxler, 2000; Qi & Mitchell, 2012), but some deaf readers do achieve high levels of reading skill (Sehyr & Emmorey, 2022; Mayberry et al., 2011). Research with these skilled deaf readers has determined that they have a different reading profile compared to hearing readers, which is characterized by comparable orthographic and semantic sensitivity without automatic recruitment of phonological codes (Bélanger et al., 2012; Emmorey & Lee, 2021). Morphological awareness (MA) is therefore an ideal candidate for study with skilled deaf readers, for whom phonology is a relative weakness, but orthographic sensitivity is a strength.

There have been a few studies that have measured deaf children and adults’ morphological awareness skills (Clark et al., 2011; Gaustad et al., 2002; Gaustad, 2004; Trussell & Easterbrooks, 2017). For example, Clark et al., (2011) implemented a morphological awareness task with 50 deaf college students who were asked to match morphologically complex words with possible meanings. The students were instructed to use “decoding” strategies to break down unfamiliar or novel words, i.e., identifying parts of the word that they did recognize to give them hints as to the novel word’s meaning. The task included both mono- and multimorphemic words. Participants also completed a phonological awareness test. Performance on the morphological awareness test, but not the phonological awareness test, was significantly related to the students’ English ment level that was assigned upon entry to Gallaudet University (Developmental, Entry Level, Advanced, or Honors). For the students in the higher levels of English ment, morphological decoding of unfamiliar complex words was an effective strategy leading to greater success in determining novel word meanings based on familiar morphemes.

Importantly, Clark et al., (2011) did not assess students’ performance on other reading skills, such as spelling or vocabulary, which could interact with morphological skills. Further, other studies investigating the factors that predict reading comprehension in deaf readers have not included a measure of morphological awareness (e.g., Cates et al., 2022; Sehyr & Emmorey, 2022). If deaf readers’ morphological awareness contributes to reading skill over and above other related skills, it could indicate that these readers rely heavily on the specific decoding process that relates to morpho-semantic segmentation. The present study aimed to investigate the relationships between morphological awareness ability as well as other reading sub-skills (spelling ability and vocabulary size) for a group of skill-matched deaf and hearing readers.

We predicted that morphological awareness would have a stronger association with reading comprehension for deaf compared to hearing readers, indicating that this skill plays a more important role in reading for deaf readers. We also conducted a linear regression analysis to characterize the relative contributions of these different reading sub-skills to reading comprehension ability. We predicted that morphological awareness would account for more variance in the deaf group’s reading comprehension than the hearing group, indicating that morphological awareness contributes to reading skill over and above spelling and vocabulary. Following Kotzer et al., (2021), we also predicted that spelling and vocabulary skill would predict morphological awareness. Furthermore, following other recent studies (Sehyr & Emmorey 2022; Mayberry et al., 2011; Clark et al., 2011; Cates et al., 2022), we predicted that phonological awareness would not predict morphological skill or reading comprehension for deaf readers, but it might be a predictor for the hearing group.

## Method

### Participants

This study included a total of 80 participants: 40 deaf adults (Mean age = 35.76, standard deviation (SD )= 8.25) and 40 hearing adults (Mean age = 29.65, SD = 1.89). Deaf participants were all prelingually and profoundly deaf and reported using American Sign Language (ASL) as a primary means of communication. All reported being exposed to ASL before age 7 (mean age of ASL exposure = 1.5 years: SD = 2.6) and reported no reading or learning disabilities. Deaf participants reported an average of 5.88 years of post-high school education (SD = 3.18). Hearing participants were all native English speakers with no reading or learning disabilities. Hearing participants reported an average of 3.7 years of post-high school education (SD = 1.63).

### Assessments

The data included in this study were compiled from an existing database of participants who completed the assessments as part of a larger battery of language and cognitive assessments being conducted for other projects (not reported here). All assessments were administered in person, in ASL or English, as appropriate for each group. Participants were tested individually. All participants took all assessments with the exception of the Phonemic Awareness Test, which was a more recent addition to the assessment battery; as such, scores for this assessment are available for only a subset of participants.

#### Woodcock Johnson IV passage comprehension subtest (LaForte et al., 2014)

Participants were asked to read short passages (1–2 sentences) containing one blank and to fill in the blank with the correct missing English word. Both deaf and hearing participants wrote out their answers (i.e., no verbal response was required). Spelling errors were not counted. Raw scores were calculated by subtracting errors from the ceiling item.

#### Test of receptive spelling (Andrews & Hersch, 2010)

Participants were given a list of 87 printed words (the word “behaviour” from the original test was excluded due to the conflict with American spelling). Some of the words were spelled incorrectly, and participants were asked to circle only the incorrectly spelled items. Raw score was calculated by subtracting errors (i.e., missed “incorrect” words or falsely circled “correct” words) from the total number of words (87).

#### Peabody picture vocabulary test IV adapted for deaf individuals (Dunn & Dunn, 2007; Sarchet et al., 2014)

In the adapted version of the peabody picture vocabulary test IV (PPVT-IV), participants read an English word in the center of a page and are asked to identify which of four pictures on the same page is the most accurate representation of the English word. Both deaf and hearing participants completed the PPVT in this adapted format, rather than the standard orally administered format. All participants began at item 157 (set 14) out of a total of 228 items (the starting point for adults). The test was discontinued when participants reached a ceiling of eight or more errors in a set. Raw scores were calculated by subtracting errors from the ceiling item.

#### Morphological awareness tests (Bernstein et al., 2020; McCutchen & Logan, 2011)

MA was assessed with two measures: the Modified Test of Morphological Structure (MTMS) and the Nonword Choice Task. Raw scores were calculated by subtracting errors from the total number of items (48). See Table 1 for example items.

**Table 1:** Example items from MTMS and Nonword Choice Tasks that comprised our measure of Morphological Awareness.

*Task*	*Example* item	*Correct Answer*
Modified Test of Morphological Structure (Part 1)	Assist. The teacher will give you ______.	Assistance
Modified Test of Morphological Structure (Part 2)	Discussion. The friends have a lot to _____.	Discuss
Nonword Choice Task	On the property was a PERIMETOUS wall. Answer options:— Encircling— Deteriorating— Rough stone	Encircling

##### Modified Test of Morphological Structure (Bernstein et al., 2020)

In Part 1 (Derivation), participants were provided with a simple root word and a sentence containing a blank and were asked to derive a complex word using the root that fits a sentence frame. In part 2 (Decomposition), participants were provided with a complex word and another sentence frame that contained a missing word and were asked to remove an affix or affixes from the complex word to produce the simple word that successfully completes the sentence. Spelling did not count as long as the participant’s answer consisted of the correct stem and the correct affix or affixes (for example, if the target item was *assistance*, *assistence* would be an acceptable answer, but *assistent* would not). There were 15 items in each part.

##### Nonword Choice Task (McCutchen & Logan, 2011)

Participants were provided with a sentence containing one orthographically plausible nonword that contained possible English affixes (e.g., acquitation) and were asked to choose from three options to identify the most plausible meaning for the nonword. There were 18 total items.

#### Phonemic awareness (PA) test (Miller et al., 1997)

A subset of the participants (26 deaf, 16 hearing) took this phonological awareness assessment as part of other ongoing projects. Participants were first familiarized with the drawings that represented the one-syllable English words on the test (i.e., star, crown, dice). In the first part of the test (six items), they were shown four of the drawings and asked to determine which two words began with the same sound (for example, *nose* and *knife*). In the second part (six items), they were asked to determine which two words ended with the same sound (for example, *horse* and *bus*). Participants were given 30 s to respond to each question. Raw score was calculated by subtracting errors from the total number of items (12).

### Analyses

Following Sehyr & Emmorey (2022), we first conducted separate correlation analyses for deaf readers (n = 40) and hearing readers (n = 40) that included reading comprehension [Woodcock Johnson (WJ) score] and the reading sub-skills: spelling (receptive spelling scores), vocabulary (PPVT scores), and morphological awareness (sum of Derivation, Decomposition and Nonword Choice scores). To compare the correlation coefficients between the two groups, we calculated the Pearson correlation coefficients for each pair of variables in both groups, resulting in two correlation matrices and Bonferroni-corrected *p*-values for each correlation.

We then assessed the contribution of reading sub-skills to morphological awareness for each group using linear regression, with morphological awareness as the outcome variable and group (deaf, hearing), spelling, and vocabulary as predictor variables (all continuous variables z-scored).

We then ran a regression model with reading comprehension as the outcome variable. The predictor variables were group, vocabulary, spelling, morphological awareness, and the interaction between group and each reading sub-skill (all continuous variables z-scored).

Finally, for the subset of participants for whom phonological awareness test scores were available (26 deaf, 16 hearing), we ran versions of the morphological awareness and reading comprehension models that also included phonological awareness and the interaction between group and phonological awareness as predictor variables (all continuous variables z-scored).

## Results

Deaf and hearing participants were matched on reading comprehension ability (WJ scores), but the groups differed on reading sub-skills. The deaf readers were significantly better spellers, while the hearing readers had larger vocabularies and scored higher on the morphological awareness tests. The descriptive statistics for each group are shown in Table 2. A subset of participants (26 deaf, 16 hearing) also had a phonological awareness score available; the hearing readers performed significantly better at this task (Deaf: M = 6.15, SD = 2.97; Hearing: M = 1.38, SD = 1.41; *p* < .01).

**Table 2:** Descriptive statistics of mean raw scores on reading assessments. Standard deviations are reported in parentheses. MA = morphological awareness.

	*Reading Comprehension*	*Spelling*	*Vocabulary*	*MA (Total)*	*MA- Derivation*	*MA- Decomposition*	*MA- Nonword Choice*
Deaf	36.98 (3.76)	74.78 (7.27)	199.12 (14.73)	29.23 (5.72)	7.3 (2.2)	9.98 (2.7)	11.83 (2.1)
Hearing	37.95 (2.52)	71.52 (8.53)	206.55 (9.18)	33.48 (4.37)	8.2 (2.07)	11.95 (1.71)	13.33 (2.19)
*p*-value	0.18	0.07	0.01	<0.01	0.06	<0.01	<0.01

### Correlation matrices

**Figure 1:**
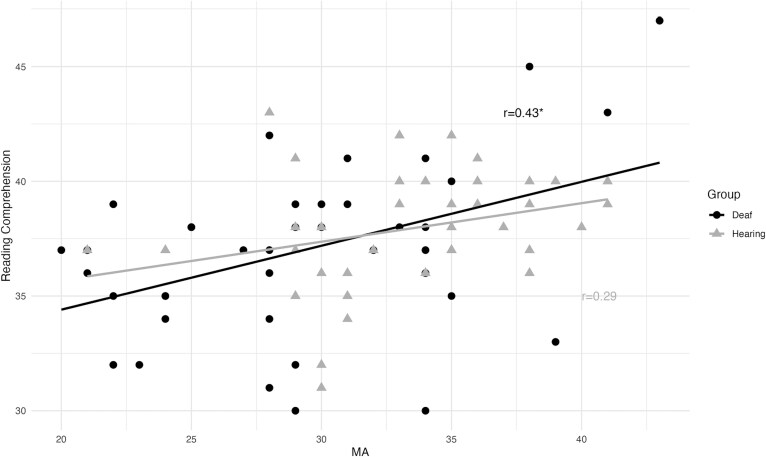
Association between morphological awareness (MA) (summed subtest scores) and reading comprehension (WJ score) for deaf and hearing readers.

As shown in Table 3, the deaf group had significant positive correlations between reading comprehension and each of the reading sub-skills. For the deaf group, morphological awareness ability was significantly correlated with spelling and marginally correlated with vocabulary, but only spelling was marginally correlated with morphological awareness for the hearing group. Figure 1 illustrates the stronger correlation between morphological awareness and reading comprehension for deaf readers compared to hearing readers.

**Table 3:** Correlations among reading sub-skills (** = Bonferroni-corrected *p* < .05, * = Bonferroni-corrected *p* < .09).

	*Reading Comprehension*	*Spelling*	*Vocabulary*	*Morphological Awareness*	
	Deaf Readers				
*Reading Comprehension*	1.00				
*Spelling*	0.41**	1.00			
*Vocabulary*	0.42**	0.46**	1.00		
*Morphological Awareness*	0.43**	0.44**	0.40*	1.00	
	Hearing Readers				
*Reading Comprehension*	1.00				
*Spelling*	0.44*	1.00			
*Vocabulary*	0.32	0.32	1.00		
Morphological Awareness	0.29	0.38*	0.25	1.00	

### Morphological awareness model

In the regression model predicting morphological awareness, we found a main effect of group, such that the hearing readers scored higher on the morphological awareness assessments (*p* = .002). Spelling skill was a significant predictor of morphological awareness for both groups (*p* = .01), and there were no interactions with group (see Table 4).

**Table 4:** Predictors of morphological awareness.

	*Morphological Awareness*					
*Predictors*	*Estimates (B)*	*SE*	*std. Beta*	*95% CI*	*t*	*p*
Intercept	31.35	0.61	0.00	30.15–32.56	51.80	*<0.001*
Group	3.97	1.21	0.72	1.56–6.39	3.28	*0.002*
Spelling	1.63	0.62	0.30	0.39–2.87	2.62	*0.011*
Vocab	1.11	0.66	0.20	−0.21 to 2.42	1.68	0.098
Group*Spell	−0.37	1.25	−0.07	−2.85 to 2.11	−0.30	0.768
Group*Vocab	−0.19	1.32	−0.03	−2.82 to 2.44	−0.14	0.885
Spell*Vocab	0.18	0.50	0.03	−0.81 to 1.18	0.36	0.718
Group*Spell* Vocab	1.13	1.00	0.21	−0.86 to 3.12	1.13	0.261
Observations	80					
R^2^/R^2^ adjusted	0.346/.282					

### Reading comprehension model

In the regression model predicting reading comprehension from the reading sub-skills, we found that spelling was a significant predictor of reading comprehension for both groups (*p =* .02), such that better spellers were stronger readers overall. There were no other main effects or interactions (see Table 5). Given our a priori hypothesis, however, we conducted additional separate regressions for each group.

**Table 5:** Linear regression predicting reading comprehension with group and reading sub-skills. MA = morphological awareness.

	*Reading Comprehension*					
*Predictors*	*Estimates (B)*	*SE*	*std. Beta*	*95% CI*	*t*	*p*
Intercept	37.58	0.39	0.04	36.80–38.36	96.50	*<0.001*
Group	0.40	0.78	0.13	−1.15 to 1.96	0.52	0.606
Vocab	0.70	0.41	0.22	−0.11 to 1.52	1.72	0.089
Spelling	0.80	0.38	0.25	0.03–1.56	2.08	*0.041*
MA	0.63	0.40	0.19	−0.18 to 1.43	1.56	0.124
Group*Spell	0.00	0.77	0.00	−1.53 to 1.53	0.00	0.999
Group*MA	−0.51	0.80	−0.16	−2.11 to 1.09	−0.63	0.529
Group*Vocab	−0.13	0.82	−0.04	−1.76 to 1.51	−0.15	0.879
Observations	80					
R^2^/R^2^ adjusted	0.286/.216					

**Figure 2:**
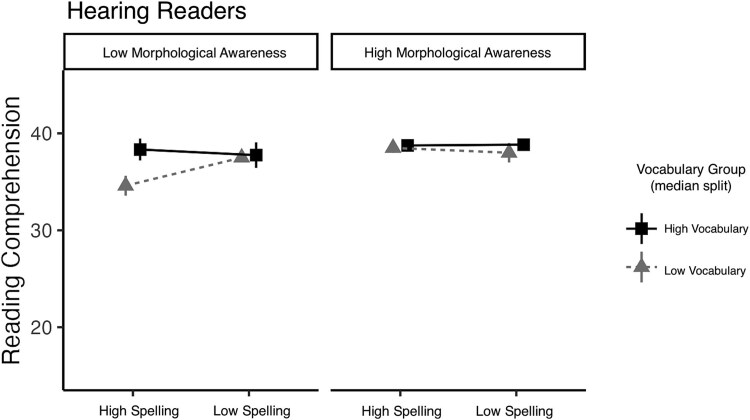
Visualization of the spelling, vocabulary, and morphological awareness interaction for the hearing readers.

For hearing readers, the model with the predictors spelling, vocabulary, morphological awareness, and the interactions among them explained 38.6% of the variance (adjusted R^2^ = .251). There was a marginal three-way interaction between spelling, vocabulary, and morphological awareness (*p* = .06); see Table 6. To visualize this interaction, we organized the data by median splits for morphological awareness skill (low, high), spelling skill (low, high), and vocabulary skill (low, high) as shown in Figure 2. For hearing readers with poorer morphological awareness and smaller vocabularies, spelling ability was associated with reading comprehension (better spellers were better readers). For those with good morphological awareness and larger vocabularies, there was no relationship between spelling skill and reading comprehension.

**Table 6:** Predictors of reading comprehension for the hearing group. MA = morphological awareness.

	*Reading Comprehension Predictors (Hearing)*					
*Predictors*	*Estimates (B)*	*SE*	*std. Beta*	*95% CI*	*t*	*p*
Intercept	37.71	0.45	0.02	36.80–38.62	84.59	*<0.001*
Spelling	0.41	0.41	0.17	−0.43 to 1.24	0.99	0.329
Vocab	0.58	0.60	0.01	−0.65 to 1.82	0.97	0.342
MA	0.92	0.55	0.16	−0.20 to 2.04	1.67	0.105
Spell*Vocab	−0.39	0.42	0.11	−1.24 to 0.47	−0.92	0.366
Spell*MA	−0.29	0.62	0.09	−1.55 to 0.97	−0.47	0.644
Vocab*MA	−1.25	0.82	−0.37	−2.92 to 0.41	−1.53	0.136
Spell*Vocab* MA	1.90	0.99	0.46	−0.12 to 3.92	1.91	*0.065*
Observations	40					
R^2^/R^2^ adjusted	0.386/.251					

**Figure 3:**
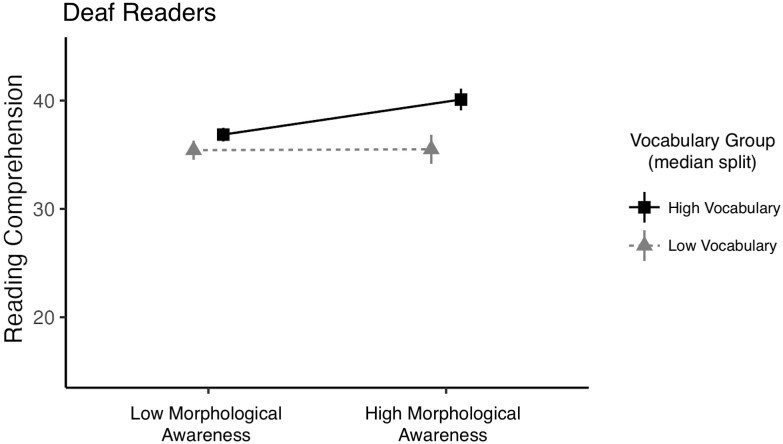
Interaction between vocabulary and morphological awareness for deaf participants.

For deaf readers, the model with the same predictors (spelling, vocabulary, morphological awareness, and the interactions among them) explained 45.5% of the variance (adjusted R^2^ = .336), a better model fit than the hearing model (Table 7). Those with larger vocabularies were stronger readers overall (*p =* .01), and there was a marginal effect of spelling, such that better spellers tended to be stronger readers (*p* = .07). There was also a significant interaction between vocabulary and morphological awareness (*p =* .03), such that deaf readers with large vocabularies and high morphological awareness were better readers than those who had large vocabularies but low morphological awareness (see Figure 3).

**Table 7:** Predictors of reading comprehension for the deaf group. MA = morphological awareness.

	*Reading Comprehension Predictors (Deaf)*					
*Predictors*	*Estimates (B)*	*SE*	*std. Beta*	*95% CI*	*t*	*p*
Intercept	36.97	0.62	−0.15	35.70–38.23	59.56	*<0.001*
Spelling	1.09	0.68	0.27	−0.31 to 2.48	1.59	0.123
Vocab	1.78	0.60	0.38	0.55–3.01	2.95	*0.006*
MA	0.96	0.69	0.21	−0.44 to 2.37	1.39	0.173
Spell*Vocab	−0.74	0.55	−0.16	−1.85 to 0.37	−1.35	0.186
Spell*MA	0.33	0.64	0.11	−0.97 to 1.62	0.52	0.609
Vocab*MA	1.07	0.50	0.31	0.04–2.10	2.12	*0.042*
Spell*Vocab*MA	−0.43	0.31	−0.12	−1.06 to 0.20	−1.40	0.172
Observations	40					
R^2^/R^2^ adjusted	0.455/.336					

**Figure 4:**
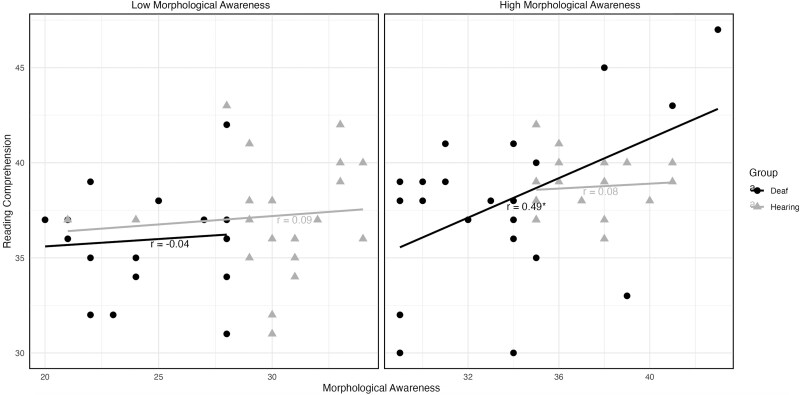
Correlations between morphological awareness and reading comprehension, split by hearing status and MA skill group.

The correlation analyses and the separate group regressions suggested that deaf readers have a different relationship with morphology than hearing readers, although the full regression model (Table 5) indicated no significant interaction between group and morphological awareness. We speculated that the differential relationship between morphological awareness and reading comprehension may have been obscured when the data were averaged across the whole range of morphological awareness skills. To test this hypothesis, we conducted additional exploratory analyses. First, we organized the dataset by morphological awareness score based on a median split of each group. We then calculated correlations between morphological awareness and reading comprehension for each skill group. There was only a significant correlation for the deaf group with high morphological awareness skill (*p* = .02) (see Figure 4).

### Phonological awareness subgroup—morphological awareness model

**Figure 5:**
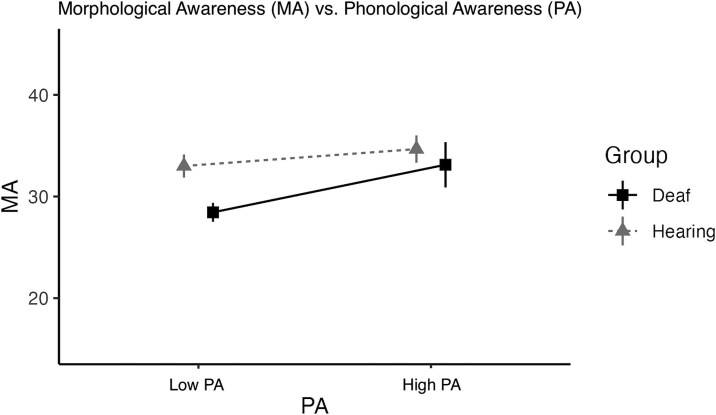
Relationship between phonological and morphological awareness.

Finally, we examined the contribution of phonology for the subset of our participants for whom a phonological awareness score was available (26 deaf, 16 hearing); see Table 8. Better spellers had higher morphological awareness for both groups (*p* = .004), as expected from the full group analysis (Table 4). Somewhat surprisingly, *both* deaf and hearing readers with higher phonological awareness also had higher morphological awareness (*p* = .002). There was also a marginal interaction between group and phonological awareness (*p =* .089), such that the association between phonological awareness and morphological awareness was stronger for the deaf readers than the hearing readers (see Figure 5). Note, however, that while both deaf and hearing groups with high phonological awareness (median split) had comparable scores on the phonological awareness test (Deaf: M = 1.13, SD = 2.03; Hearing: M = 11.44, SD = .52), the low-scoring hearing group had nearly double the score of the low-scoring deaf group (Deaf: M = 4.6, SD = .91; Hearing: M = 9.0, SD = .81) and the hearing had a considerably smaller range (8–12) than the deaf group range (0–12).

**Table 8:** Predictors of morphological awareness for the subgroup with phonological awareness (PA) scores.

	*Morphological Awareness*					
*Predictors*	*Estimates (B)*	*SE*	*std. Beta*	*95% CI*	*t*	*p*
Intercept	30.59	0.92	−0.10	28.73–32.46	33.33	*<0.001*
Group	4.62	2.39	0.92	−0.22 to 9.46	1.94	0.061
Vocab	0.30	0.66	0.06	−1.04 to 1.64	0.46	0.649
Spelling	2.29	0.75	0.40	0.77–3.81	3.05	*0.004*
PA	2.77	0.82	0.55	1.10–4.44	3.37	*0.002*
Group*PA	−4.20	2.40	−0.84	−9.07 to 0.68	−1.75	*0.089*
Observations	42					
R^2^/R^2^ adjusted	0.498/.428					

### Phonological awareness subgroup—reading comprehension model

For the hearing group, phonology was the only significant predictor of reading comprehension (*p* = .004). For the deaf group, better spellers were marginally better readers (*p* = .07) and those with larger vocabularies were better readers (*p* = .03). Phonological awareness did not impact reading comprehension for the deaf group (see Table 9).

**Table 9:** Predictors of reading comprehension including phonological awareness (PA) for subgroup of participants (MA = morphological awareness).

	*Predictors of Reading Comprehension*					
*Hearing*						
*Predictors*	*Estimates (B)*	*SE*	*std. Beta*	*95% CI*	*t*	*p*
Intercept	34.92	1.20	−0.00	32.27–37.56	29.09	*<0.001*
Spelling	−0.59	0.65	−0.25	−2.02 to 0.83	−0.92	0.379
Vocab	0.52	0.62	0.17	−0.86 to 1.89	0.83	0.426
PA	3.94	1.11	0.80	1.51–6.38	3.57	*0.004*
MA	0.30	0.83	0.09	−1.52 to 2.13	0.36	0.723
Observations	16					
R^2^/R^2^ adjusted .574/.419						
*Deaf*						
*Predictors*	*Estimates (B)*	*SE*	*std. Beta*	*95% CI*	*t*	*p*
Intercept	37.73	0.82	0.00	36.03–39.43	46.17	*<0.001*
Spelling	1.72	0.90	0.34	−0.16 to 3.60	1.90	0.071
Vocab	1.47	0.66	0.37	0.10–2.84	2.24	*0.036*
PA	0.92	0.84	0.21	−0.83 to 2.67	1.10	0.285
MA	0.52	0.92	0.12	−1.39 to 2.43	0.57	0.577
Observations	26					
R^2^/R^2^ adjusted	0.464/.362					

## Discussion

This study investigated predictors of reading comprehension for a group of similarly skilled deaf and hearing readers, with a focus on the impact of morphological awareness. We first conducted correlation analyses to characterize the relationships among the various sub-skills of reading: spelling, vocabulary size, and morphological awareness. This analysis was followed up with a regression model predicting morphological awareness from these sub-skills, then a regression model predicting reading comprehension. Finally, we analyzed the subgroup of participants for whom a phonological awareness score was also available, including phonological skill in the model predicting morphological awareness and then reading comprehension.

We hypothesized that the importance of orthographic-to-semantic mappings for deaf readers would result in a stronger reliance on morphological information for word comprehension. Deaf readers should therefore show a more pronounced relationship between morphological awareness and reading comprehension skill. Our findings from the correlation analyses supported this hypothesis. MA had a stronger positive relationship with reading comprehension for deaf than hearing readers, suggesting that morphology may be a stronger contributor to skilled reading for deaf readers. Furthermore, the finding that morphological awareness was more strongly related to vocabulary size for deaf readers than for hearing readers indicates that morphological awareness may specifically impact how deaf readers expand their vocabularies. We suggest that deaf readers may be using familiar morphemes to conduct “morphological problem solving” and infer or estimate meaning from complex or unfamiliar words, a process also observed for hearing students (Carlisle, 2000). This interpretation is consistent with the findings of Clark et al. (2011) who found that deaf college students who were better able to use morphology-based strategies to understand novel words were stronger English readers.

The regression model revealed a relationship between morphological awareness and reading comprehension skill for deaf readers that was not observed for hearing readers and vice versa. Specifically, for deaf readers, morphological awareness was a stronger predictor of reading skill for those with larger vocabularies, again supporting the idea that morphology and vocabulary are uniquely intertwined for deaf readers. For hearing readers, spelling ability was more strongly associated with reading comprehension (better spellers were better readers), and this pattern was stronger for hearing readers with poorer morphological awareness and smaller vocabularies. Thus, sensitivity to orthographic form (spelling) is more beneficial for hearing readers with poorer reading subskills (including morphology), whereas for deaf readers sensitivity to morphology is more beneficial to reading success, but primarily for those who know more English words.

In separate group regressions models, deaf readers again appeared to have a different relationship to morphology than hearing readers. Based on the visualization of the correlation data (Figure 1), we hypothesized that morphology may be more important for deaf readers who are highly familiar with morphological structure. To test this hypothesis and further characterize this relationship, we calculated the correlations between morphological awareness and reading comprehension for each group, separated by morphological awareness skill (Figure 4). Of the four subgroups, only the deaf readers with high morphological awareness had a strong positive relationship between morphology and reading comprehension. The group of deaf readers with low morphological awareness did not have a significant relationship between the two measures, nor did either group of hearing readers. This result indicates that once deaf readers reach a certain level of morphological proficiency, morphological awareness can play a strong role in reading comprehension. This finding is consistent with previous research showing that deaf college students with higher English ment levels were more successful on a morphological decoding task (Clark et al., 2011).

Highly skilled deaf readers with a strong grasp of morphology may be leveraging the visual accessibility of morpho-orthographic segments to decompose unfamiliar complex words when they encounter them, and thus boost their overall comprehension abilities. This hypothesis is consistent with the fact that morphologically complex words make up a majority of the words encountered by readers above middle school reading levels (Nagy & Anderson, 1984). For the skilled deaf readers, complex words may be identified through morphographic segmentation of component morphemes, rather than phonemic decoding (Gaustad, 2000). Hearing readers, despite having high morphological awareness, did not show a significant relationship between reading comprehension and morphology, indicating that this process may be less important to this group of readers. However, this null result should be interpreted with caution given our relatively small sample size and previous results indicating that morphological skill predicts reading ability in hearing adults (e.g., Kotzer et al., 2021).

Phonological awareness has long been considered one of the most important factors in reading success; however, it has been less studied in relation to morphological awareness. Phonological awareness was a strong predictor of morphological awareness for both deaf and hearing readers, with the deaf readers showing a stronger association. However, the much smaller range of scores on the phonological awareness test for the hearing readers could have reduced the strength of the association with morphological awareness for this group. Nonetheless, phonological awareness was a strong predictor of reading comprehension for the hearing readers, whereas it was not a predictor for the deaf group, replicating previous studies (Cates et al., 2022; Mayberry et al., 2011; Sehyr & Emmorey, 2022). The finding that deaf readers with better phonological awareness had better morphological skills is novel and somewhat surprising, given the lack of relationship between phonological skills and reading comprehension for these readers. One speculative explanation is that these readers’ morphological awareness skill could reflect overall higher metalinguistic awareness of sublexical components of print. This higher awareness could result in stronger links between orthography and phonology, as well as the link between orthography and meaning. As proposed by Kirby and Bowers (2017, 2018), morphemes may act as binding agents, providing information about not only semantics, but also phonology and orthography. Thus, deaf readers who have stronger morphological representations may also have stronger phonological skills and stronger semantic knowledge (as evidenced by the positive correlation between vocabulary size and morphological awareness).

It is important to note that the hearing group was more skilled at morphological awareness overall, with significantly higher scores on the morphology assessments, despite the groups being matched on reading comprehension. This result could be due to the fact that the deaf group, all of whom reported ASL as their native language, were all reading in their second language (English). In contrast, the hearing readers were all monolingual and reading in their native language (see Limitations for additional discussion). MA for bilinguals is known to be affected by the reader’s L1 (Wu & Juffs, 2022), and while ASL does have a rich morphological system (Aronoff et al., 2005), little is known about the influence of ASL morphology on English morphological awareness. Much of ASL morphology involves non-concatenative morphological processes, such as reduplication, rather than the addition of prefixes and suffixes to a stem as in English. Thus, although the knowledge that complex word forms can be analyzed into meaningful parts may transfer from ASL to English, there are few translation-equivalent morphemes (e.g., −ly, −ion), and word creation processes differ between ASL and English.

### Limitations & future directions

Due to the exploratory nature of this study, one limitation is that some of our effects were post-hoc follow up analyses based on the initial planned analyses. For example, we did not find a significant group by morphological awareness interaction in the full regression model, but our follow up regressions revealed different patterns of predictors in separate analyses of the deaf and hearing groups. In addition, our phonological awareness test may not have been sensitive enough for the hearing readers, given the small range of scores for this group. The analysis of the phonological awareness subgroup should also be interpreted with caution, given that the inclusion of the additional factor could impact the reliability of the results.

Another potential limitation is that we compared hearing English monolinguals with deaf ASL-English bilinguals. Although monolingual hearing readers have long been a de facto comparison group, recent research has suggested that deaf readers’ bilingualism may play an important role in potential reading differences, thus motivating a comparison group of bilingual speakers. For example, Cates et al. (2022) compared hearing Chinese-English bilinguals with deaf ASL-English bilinguals to account for possible bilingualism effects on reading. However, use of a bilingual comparison group is not without issues because deaf bilinguals, in contrast to most hearing bilinguals, read printed text in only one language, as ASL does not have a standard written form. While hearing bilinguals may divide their reading time between two different languages, deaf bilinguals, like hearing monolinguals, do not. Therefore, deaf readers constitute a unique type of bilingual that still shares certain characteristics with hearing monolingual readers. For the current study, the shared experience of reading consistently in one language only was the primary motivation for using hearing monolingual readers as a comparison group. To further investigate this issue, future research could include bilinguals who are only literate in one of their languages, such as heritage bilinguals who speak a home language, but do not read or write in that language.

The finding that morphology is related to reading comprehension for skilled deaf readers provides support for proposals that explicit morphological instruction should be incorporated into literacy programs for deaf students. The relatively transparent orthographic-to-semantic mapping and regular structure of the English morphological system may be a more accessible and efficient pathway to word learning and reading comprehension, compared to opaque sound-to-meaning mappings. Small-scale but successful intervention studies have illustrated that morphographic analysis of spelling cues is a route through which deaf students can ascertain the meaning of new and complex words (Trussell, 2020; Trussell et al., 2018; Trussell & Easterbrooks, 2015). If explicit morphological instruction can boost deaf readers’ morphological awareness skills, they may be able to leverage those skills to improve their reading comprehension overall. By specifically targeting morphological awareness, teachers and other deaf education professionals may be able to use the more accessible route to reading comprehension to help close the achievement gap experienced by many deaf students. Future research should also further investigate the relationship between morphological and phonological awareness in deaf readers to identify the direction of the effects, e.g., whether morphological skill improves phonological knowledge or vice versa.

## Conclusion

This study characterizes the reading profiles of two groups of similarly skilled deaf and hearing readers, focusing on the role of morphological awareness. Overall, we demonstrated that morphological awareness has a stronger relationship with reading comprehension for deaf readers than it does for hearing readers. Deaf readers likely utilize the accessibility of orthographically defined morphemes to infer novel word meanings and expand their vocabularies, suggested by the links between morphological awareness and vocabulary size. While hearing readers were generally more skilled at morphological awareness, deaf readers with high morphological skills demonstrated a stronger link between morphology and reading comprehension, indicating that this strategy was specific to deaf readers, rather than being a consequence of stronger morphology skills in general. Overall, the findings highlight the unique and significant role of morphological awareness in the skilled deaf reader’s “toolbox.” Expanding our knowledge of morphological processing in deaf readers is crucial for the development of more effective reading interventions that tap into their unique linguistic potential.
